# Liposomal Copermeation Assay Reveals Unexpected Membrane Interactions of Commonly Prescribed Drugs

**DOI:** 10.1021/acs.molpharmaceut.3c00766

**Published:** 2024-04-29

**Authors:** Klára Odehnalová, Martin Balouch, Kateřina Storchmannová, Eliška Petrová, Magdalena Konefał, Aleš Zadražil, Karel Berka, Jiří Brus, František Štěpánek

**Affiliations:** † Department of Chemical Engineering, University of Chemistry and Technology Prague, Technická 5, Prague 6 166 28, Czech Republic; ‡ Zentiva, k.s., U Kabelovny 130, Prague 10 102 37, Czech Republic; § Department of Physical Chemistry, Faculty of Science, Palacký University Olomouc, 17. listopadu 12, Olomouc 771 46, Czech Republic; ∥ Department of Organic Technology, University of Chemistry and Technology Prague, Technická 5, Prague 6 166 28, Czech Republic; ⊥ Institute of Macromolecular Chemistry of the Czech Academy of Sciences, Prague 6 162 00, Czech Republic

**Keywords:** permeability, partitioning coefficient, liposomes, drug interaction, fluorescence quenching

## Abstract

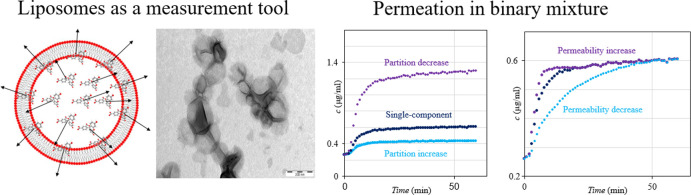

The permeation of small molecules across biological membranes is a crucial process that lies at the heart of life. Permeation is involved not only in the maintenance of homeostasis at the cell level but also in the absorption and biodistribution of pharmacologically active substances throughout the human body. Membranes are formed by phospholipid bilayers that represent an energy barrier for permeating molecules. Crossing this energy barrier is assumed to be a singular event, and permeation has traditionally been described as a first-order kinetic process, proportional only to the concentration gradient of the permeating substance. For a given membrane composition, permeability was believed to be a unique property dependent only on the permeating molecule itself. We provide experimental evidence that this long-held view might not be entirely correct. Liposomes were used in copermeation experiments with a fluorescent probe, where simultaneous permeation of two substances occurred over a single phospholipid bilayer. Using an assay of six commonly prescribed drugs, we have found that the presence of a copermeant can either enhance or suppress the permeation rate of the probe molecule, often more than 2-fold in each direction. This can have significant consequences for the pharmacokinetics and bioavailability of commonly prescribed drugs when used in combination and provide new insight into so-far unexplained drug–drug interactions as well as changing the perspective on how new drug candidates are evaluated and tested.

## Introduction

Membrane permeability and water/membrane partitioning coefficient are two key parameters determining the biodistribution and bioavailability of pharmaceutically active substances in living organisms. They affect the absorption of a drug upon administration (oral, transdermal, and inhalation), its subsequent distribution in the body, and accumulation in individual organs and tissues. Biological membranes consist of phospholipid bilayers enriched with a diverse array of proteins and saccharides, carrying out various functions from signaling to transport. Transmembrane proteins responsible for actively transporting molecules are of utmost importance, with their impact on the coadministration of molecules, especially *p*-glycoprotein or anion-transporting polypeptides.^1−3^ Equally important to active transport is a passive one. The rate at which a given molecule permeates across a membrane depends on the energy barrier represented by the phospholipid bilayer. The structure of the lipid bilayer can be influenced by the presence of other nonpermeating molecules. This phenomenon is called permeability enhancement and has been studied extensively with regard to skin^4−6^ or intestinal^7^ permeability.

Examples of simple permeation enhancers include ethanol, oleic acid, or dimethyl sulfoxide, but new enhancers and enhancement mechanisms are being actively investigated.^8,9^ An opposite phenomenon–permeation retardation remains rather unexplored, although its biological and pharmacological implications can be just as important.^10^ The ability to suppress the permeation rate of specific compounds could, for example, enable previously rejected drugs, which were found to be too “leaky” and thus unsuitable for liposomal formulation^11^ to be revisited. Not being aware of permeation enhancement or permeation suppression caused by a medicinal substance that was not *a priori* meant to do so could be problematic, especially in the context of the so-called polypharmacy patients, who are simultaneously prescribed by many (typically five or more) medicines simultaneously.

The permeability of a substance across a membrane of a given composition has been traditionally assumed to depend only on the properties of the molecule itself (charge, lipophilicity, molar weight, etc.). In textbooks, permeation is described as a first-order kinetic process, proportional only to the concentration gradient of the permeating molecule alone. Experimental and computational permeation results have so far been interpreted in a way that assumed permeability to be a unary property. However, there is an increasing body of scientific literature pointing at potential drug–drug interactions in polypharmacy patients, many of whom are systematically over- or under-dosed due to significantly different bioavailability profiles when some drugs are prescribed in combination rather than alone.^12^

Interestingly, such interactions were reported even for drugs that target very different metabolic pathways and that should not, in theory, influence each other at the molecular target level. These phenomena could potentially be explained by considering permeability a binary (or higher order) property, i.e., by considering that the permeation rate of molecule A could also depend on the concentration of molecule B (or C, etc.). However, no direct experimental evidence for such collective permeation properties has been available so far, and in fact, there was no method for reliably measuring copermeation.

Experimental methods for studying membrane permeability and partitioning typically rely on measuring the concentration change of a single permeant in two macroscopic reservoirs separated by a planar membrane model. The permeation barrier can be formed synthetically from lipidic materials as in the PAMPA assay,^13^ assembled from living cells as in the Caco-2 permeability method,^14^ or collected from real tissues such as skin in the Franz diffusion cells.^5^ The interpretation and cross-laboratory comparison of data obtained by the above-mentioned methods are complicated by the fact that permeation typically occurs across multiple lipid bilayers, whose exact count is rarely known or reported. Another common feature of the above methods is that the permeation area is limited to a few square centimeters, which means that very long measurement times are needed in the case of low-permeability substances. Therefore, significant efforts have been devoted also to the development of computational methods for determining membrane permeability and partitioning of individual molecules.^15−18^

The problem of low surface area and an unknown number of lipid bilayers can be overcome by replacing the macroscopic planar membrane analogue with liposomes. Liposomes are spherical molecular assemblies comprising a lipid bilayer enclosing an aqueous core.^19−21^ Their size and lamellarity can be fairly well controlled.^22^ Liposomes are used as drug delivery vehicles thanks to their proven biocompatibility and tunable properties. Examples of liposome-based drug formulations include Doxil^19^ or the recent mRNA COVID-19 vaccines.^20,21^ Not all molecules are directly suitable for liposomal encapsulation.^23^ Too high or too low of a permeability prevents a drug from being reasonably retained and released from liposomes. Nevertheless, liposomes lend themselves as a tool for studying permeation and measuring permeability.^24^ Methods based on detecting a pH change induced by the permeation of a weak base into liposomes,^25^ on preloading liposomes with engineered receptors whose fluorescence is quenched by the permeating molecule,^26^ or on the so-called immobilized liposome chromatography^27−29^ have been reported.

**Figure 1:**
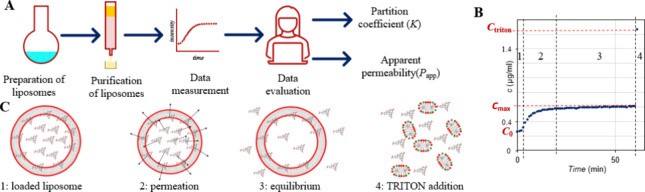
(A) Schematic representation of the liposomal copermeation method. Liposomes were preloaded with a fluorescent probe (carboxyfluorescein, CF) and a copermeant; after separating liposomes from the supernatant, the release kinetics into a fresh medium was induced by a temperature step; the release curve was evaluated by a mathematical model that provided two parameters: permeability and partitioning coefficient. These were then compared between single-component permeation and copermeation. (B) Typical result of pure CF permeation, showing four stages. Stage 1: no release at room temperature; stage 2: permeation after heating to the lipid bilayer phase transition; stage 3: equilibrium between the intra- and extra-liposomal concentration of the permeant; stage 4: dissolution of the lipid bilayer by Triton, causing the release of the membrane-bound permeant. (C) Schematic representation of phenomena that occur during each stage of the experiment.

Here, we present original copermeation experimental data obtained by means of a liposome permeation assay on a sample of six commonly prescribed drugs. The principle of the method is shown in Figure 1. Our data reveal both positive and negative interactions of copermeating molecules, providing the first direct evidence of collective permeation and partitioning behavior that could have far-reaching consequences both for the prescription practices of existing drugs and for the evaluation of new ones.

## Experimental Section

### Materials

Phosphate-buffered saline in tablets (PBS), 5(6)-carboxyfluorescein (CF, >95%), norfloxacin (NX, >98%), cholesterol (>99%), kanamycin sulfate (KM), Triton X-100 (laboratory grade), and oleic acid (OA, 90%) were purchased from Sigma-Aldrich s.r.o. Dipalmitoylphosphatidylglycerol (DPPG) and dipalmitoylphosphatidylcholine (DPPC) were purchased from Corden Pharma. Sodium hydroxide (NaOH, p.a.), ascorbic acid (ASC, p.a.), sodium chloride (NaCl, p. a.), phosphoric acid (H_3_PO_4_, >75%), and disodium hydrogen phosphate dodecahydrate (Na_2_HPO_4_·12H_2_O) were purchased from PENTA s.r.o. Chloroform (p.a.) and ethanol (EtOH, >99.8%) were purchased from Lach-Ner s.r.o., and methanol (>99.8%) was purchased from Fisher Scientific s.r.o. Hydrochlorothiazide (HCTZ), candesartan cilexetil (CC), and apixaban (APIX) were kindly provided by Zentiva k.s. All substances and materials were used as supplied and were not modified. Deionized water (Aqual 25, 0.07 μS/cm) was used in all experiments.

### Preparation of Liposomes

Liposomes were prepared by a standard lipid film hydration method. The mixture of phospholipids and cholesterol (DPPC:DPPG:cholesterol, 75:10:15, 10 mg in total) was dissolved in 10 mL of methanol:chloroform solution (1:1 by volume). Subsequently, the solvent mixture was evaporated on a vacuum rotary evaporator (60 °C, gradually reducing the pressure from atmospheric to approximately 80 mbar). This process produced a dried lipid film, which was subsequently dried in a desiccator for at least 3 h (30 mbar).

The completely dried lipid film was then hydrated with 2 mL of the aqueous medium (7.5 mg/mL carboxyfluorescein solution in PBS, pH 7.4). PBS contained 0.01 M phosphate buffer, 0.0027 M potassium chloride, and 0.137 M sodium chloride. The sample and the extruder (Avanti Mini Extruder) were heated to 69 °C for 10 min, and the sample was then vortexed to form raw liposomes. To increase the uniformity of the liposomes, the sample was extruded at least 21 times through a membrane with a pore size of 200 nm (at 69 °C).

The prepared liposomes were characterized. Particle size distribution was determined using dynamic light scattering (DLS), and the zeta potential was determined using electrophoretic light scattering (ELS) (both Malvern Zeta sizer Nano-ZS) and by images from transmission electron microscopy (TEM, Jeol JEM-1010, accelerating voltage 80 kV).

### Encapsulation of Copermeants

The hydrophilic substances (ascorbic acid and kanamycin) and the mildly soluble lipophilic substances (hydrochlorothiazide and norfloxacin) were added to the hydration medium (solution CF in PBS) during lipid film hydration (aqueous addition route). Lipophilic substances (apixaban, candesartan cilexetil, hydrochlorothiazide, and norfloxacin) were added during the first step of liposome preparation, i.e., they were mixed with the phospholipids and dissolved in a mixture of chloroform and methanol (lipid addition route). All samples were prepared in triplicates.

### Purification of Liposomes

All liposome samples were purified by size exclusion chromatography using PD Minitrap G-25 separation columns to separate the surrounding hydration solution from the liposomes themselves. In this way, 1 mL of purified liposome solution was collected. The principle of CF release kinetics measurement is based on the fluorescence quenching of concentrated CF. The intraliposomal CF does not fluoresce; its fluorescence increases sharply only upon dilution after release from the liposomes. For this reason, the hydration medium had to be separated from the liposomes before any permeation experiments. About 10% of nonencapsulated CF remained in the solution outside liposomes after column separation; this was deduced from subsequent CF release experiments as a baseline.

### High-Performance Liquid Chromatography (HPLC) Methods

Copermeanst concentrations (CC, NFX, and HCTZ) in the liposomes were determined by Vanquish reverse-phase high-performance chromatography (Thermo Fisher Scientific, USA) using Kinetex 5 μm C18 100 Å (150 × 4.6 mm; Phenomenex, USA) as the stationary phase including guard column SecurityGuard Cartridges (C18 4 × 3 mm ID, Phenomenex, USA). All methods were developed and validated in-house. The validation was performed to determine the accuracy, precision, and linearity in the range of 50–0.05 μg/mL. Data were evaluated in the software Chromeleon 7.3.1, and each method parameter was described in the following subsections.

CC: The mobile phase consisted of acetonitrile/distilled water with pH = 3 adjusted by phosphoric acid in a ratio of 80:20. The flow rate was 1 mL/min, column temperature was 40 °C, detection wavelength was 254 nm, injection volume was 20 μL, and retention time was 3.1 min.

HCTZ: The mobile phase consisted of acetonitrile/distilled water with pH = 4 adjusted by phosphoric acid in a ratio of 20:80. The flow rate was 0.8 mL/min, column temperature was 20 °C, detection wavelength was 270 nm, injection volume was 5 μL, and retention time was 3.9 min.

NX: The mobile phase consisted of methanol/acetonitrile/distilled water with pH = 3 in a ratio of 3:17:80. The flow rate was 1 mL/min, column temperature was 20 °C, detection wavelength was 285 nm, injection volume was 15 μL, and retention time was 2.5 nm.

### Permeation Measurement

From a stock of purified liposomes, 60 μL was pipetted into a disposable cuvette and mixed with 1140 μL of PBS. Then, the measurement (in triplicate for each sample) of CF permeation through the membrane was carried out in a fluorescence spectrophotometer (Cary Eclipse, Agilent) in which the sample was heated to the desired temperature (30, 40, and 50 °C), which was kept constant throughout the measurement. The following settings were used: excitation wavelength: 490 nm, emission wavelength: 522 nm, excitation slit: 2.5 and 2.5, scan control: slow, detector voltage: medium, and maximum intensity: 1000 au. The time dependence of the fluorescence intensity at constant temperatures was measured. At the end of the experiment, 5 μL of 10 times diluted Triton X-100 was added to cause total micellization of the system, thus releasing all previously unreleased CF. The mechanism of this micellization is shown in Figure 1C and is based both on the experiment^30^ and molecular dynamics study.^31^ The measured fluorescence intensity dependence on time was then converted to a CF concentration using a calibration curve (see the Supporting Information, Figure S1).

The relative amount released of CF was then determined:
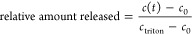
1where *c*(*t*) is the mass concentration of CF at time *t*, *c*_0_ is the CF mass concentration at the beginning of the measurement, and *c*_triton_ is the final CF mass concentration after liposome micellization by the addition of Triton X-100. The partition coefficient was calculated from the mass balance using the relation:
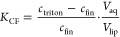
2where *c*_fin_ is the asymptotic mass concentration of CF achieved by thermal release, i.e., the final concentration at the end of the experiment just before Triton addition, *V*_aq_ is the volume of the aqueous environment, and *V*_lip_ is the volume of lipid membrane taken as
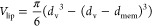
3where *d*_v_ is the volume mean diameter of liposomes evaluated from DLS measurement and *d*_mem_ is the membrane thickness, assumed to be constant and taken as 4.059 nm.^32^

Permeability was evaluated using an exponential model with a concentration driving force for the efflux. From the combination of CF mass balance inside and outside of liposomes:
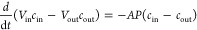
4where *V*_in_ and *V*_out_ are the volumes of the aqueous phase inside and outside of the liposomes, respectively, *c*_in_ and *c*_out_ are CF concentrations, *A* is the total surface area for permeation, and *P* is the permeability. Due to the self-quenching property of CF, only *c*_out_ is assumed to contribute to fluorescence that is experimentally measured (calculated from measured fluorescence intensity using a calibration curve). Any CF associated with the lipidic phase is also assumed not to contribute to the sample fluorescence until it is released by Triton addition at the end of the experiment.

Equation 4 can be combined with the mass balance of CF:
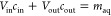
5where *m*_aq_ is the total mass of CF in the aqueous phase and is taken as the constant. After integration, the model leads to an exponential function that can be expressed as
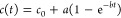
6where *a* and *b* are the regression parameters obtained from experimentally measured CF release curves. Then, the permeability coefficient is calculated as
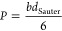
7where *d*_Sauter_ is the surface area mean diameter of the liposomes.

### Small-Angle X-ray Scattering

Small-angle X-ray scattering (SAXS) measurements were carried out to determine the lamellarity of liposomes in the presence of copermeants. The SAXS experiments were performed using a MolMet pinhole camera (Rigaku, Tokyo, Japan), upgraded by SAXSLAB (Xenocs, Grenoble, France) equipped with a vacuum version of a Pilatus 300 K detector. The camera was attached to a microfocused X-ray beam generator Rigaku MicroMax 003, operating at 0.6 mA and 50 kV. The scattering vector *q* is defined as *q* = 4π/λ·sinΘ, where λ is the wavelength (CuKα line = 1.54 Å) and 2Θ is the scattering angle. The sample-to-detector distance, calibrated using a silver behenate standard, was set up to give access to an overall *q* range of 0.004–0.65 Å^–1^. The exposure time of each measurement was 300 min. Azimuthal integration of the intensity obtained from 2D images was performed to get one-dimensional SAXS curves. The data were fitted using Sasfit software (version 0.94.11)^33^ with the log-normal function to obtain the exact position of the peaks.

### Permeation Enhancers

For the study of permeation enhancers, CF-containing liposomes were prepared and purified, as described above. For permeation enhancement by ethanol, 60 μL of purified liposomes with encapsulated CF was mixed with 1140 μL of PBS in a measuring cuvette. The samples were maintained at 30 °C. At approximately 5 min intervals, 40 μL of ethanol was added to the measuring cuvette from the top and the fluorescence intensity was measured by fluorescence spectrophotometry as described above. For permeation enhancement by oleic acid, the procedure was very similar to ethanol, only the volumes were different (50 μL of pure oleic acid, 1090 μL of PBS, and 60 μL of the CF sample purified by column chromatography), and only one addition at the start of the experiment was done. The temperature was also 30 °C.

## Results

### Single-Component Permeation Measurement by the Liposomal Assay

**Figure 2:**
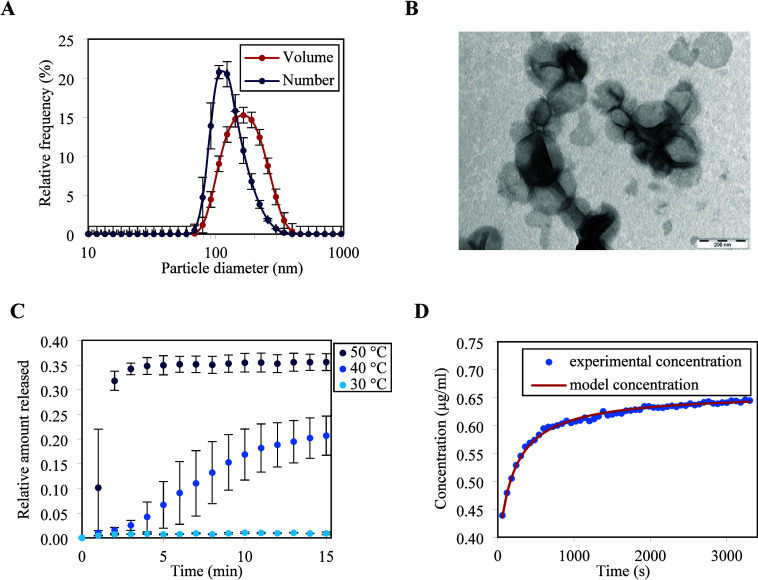
(A) Particle size distribution of liposomes with encapsulated CF, measured by dynamic light scattering. (B) TEM micrograph of the prepared liposomes. (C) Thermally induced release of encapsulated CF from liposomes at three different temperatures (the phase transition temperature of the used lipid bilayer is 41.5 °C). The data points are mean values, and error bars indicate standard deviations (*n* = 3). (D) Comparison of the CF release curve measured at 40 °C with regression by a mathematical model, which was used for evaluation permeability from the experimental data.

Dynamic light scattering (Figure 2A) and TEM (Figure 2B) analyses of purified liposomes containing encapsulated carboxyfluorescein (CF) as a fluorescent probe reveal that a population of liposomes with a mean particle size around 200 nm was prepared. The polydispersity index (PDI) of the liposomes was 0.05–0.10. At a lipid concentration of 5 mg/mL, the total surface area of such liposome is approximately 2 m^2^/mL, which represents an increase by a factor of 10^4^ compared to traditional permeation assays with planar membranes. The liposomes were colloidally stable; their zeta potential determined by electrophoretic light scattering was (−12.4 ± 1.3) mV. The negative surface charge is consistent with the fact that a negatively charged phospholipid DPPG was used as part of the membrane mix. The liposomes were predominantly unilamellar (Figure S2, Supporting Information). After column separation from nonencapsulated CF, the background concentration of CF in the aqueous phase outside liposomes decreased from 7500 to around 0.3 μg/mL. The liposome recovery after column separation was approximately 70% (based on the quantification of an assay containing fluorescently labeled lipids NBDPC), giving a final lipid concentration of approximately 3.5 mg/mL.

To utilize liposomes for permeation measurements, the temperature dependence of permeation rate had to be established first. A lipid bilayer can exist in the gel phase or in the liquid crystalline phase, which differs dramatically in their permeation properties. The phase transition temperature of the three-component lipid bilayer with cholesterol, which was used in this work, has been previously shown^34^ to be 41.5 °C. In a permeation assay, the liposomes should not be permeable at laboratory temperature, but it should be possible to start permeation by raising temperature. Three temperatures were investigated: 30, 40, and 50 °C. The experiment was run for 15 min. The time dependence of the relative amount of CF released (Figure 2C) reveals that at 30 °C, which is safely below the phase transition temperature, there was no permeation throughout the measurement period. At the other extreme at 50 °C, which is well above the phase transition temperature, permeation was too rapid, and it would be inaccurate to evaluate permeability from only a few data points. A suitable temperature thus proved to be 40 °C, which was just below the phase transition but close enough for CF permeation to already occur at a reasonable rate. The measured CF release curve (time dependence of the concentration over time taken from the inflection point onward) was regressed by an algebraic model, detailed in the Experimental section. An excellent agreement between the model and experiment was obtained (Figure 2D).

The liposomal permeability of CF in the PBS medium had a value of (1.4 ± 0.4) × 10^–8^ cm/s, which is consistent with previously reported values obtained from the COSMOPerm calculation (≈10^–8^ cm/s).^15,35^ Furthermore, the membrane/water partition coefficient was evaluated for this sample according to eq 3, which had a value of (2.9 ± 0.2) × 10^4^ (log *P* = 4.5). Here, it is important to note that this partition coefficient is unexpectedly high for a water-soluble molecule (theoretical octanol/water partitioning coefficient for CF, computed by XLogP3,^36^ is log *P* = 2.9). A possible explanation could be that CF interacts with the membrane not only by partitioning into the bilayer but also by surface adsorption. Furthermore, it should be considered that the fluorescence quenching of CF at higher concentrations is caused by the formation of CF dimers, in which the CF molecules interact with each other via polar groups.^47^ Such dimers can be expected to be less hydrophilic than CF molecules alone, increasing the partitioning coefficient of the phospholipid bilayer. To support this hypothesis (fluorescence quenching of membrane-associated CF, fluorescence dequenching after liposome micellization by the addition of Triton^37^), we have performed a liposome titration experiment (Supporting Information, Figure S4). A solution of pure liposomes was added to the CF solution in three aliquots. This led to the lowering of the fluorescence intensity, which was proportional to the quantity of added liposomes. When Triton was added, the fluorescence intensity reverted to its original value for pure CF solution. This experiment validates the assumptions behind eqs 1 and 2, which underpin the calculation of the partition coefficient.

### Direct Observation of Permeation Enhancement Mechanisms

**Figure 3:**
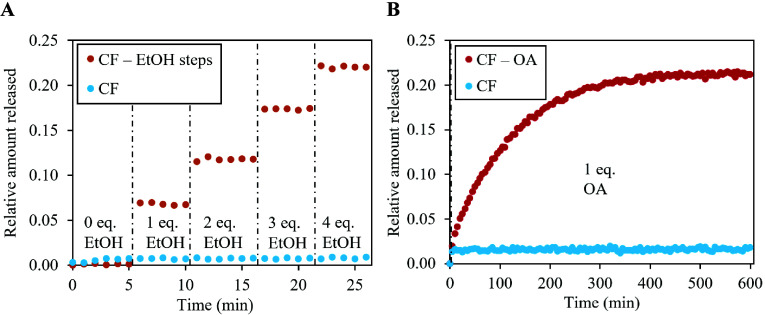
Experimentally measured dependence of the relative amount of CF released from liposomes on time at 30 °C. (A) Stepwise addition of ethanol into the system. (B) Addition of oleic acid. Note that the duration of the experiment was 600 min, in the case of oleic acid. Blue data points represent the base case (only CF), and red data points represent permeation in the presence of the permeation enhancer.

The liposomal assay employed in this work allows for direct observation of permeation enhancement in a single bilayer of phospholipids. Two well-known permeation enhancers were studied: ethanol and oleic acid. Permeation enhancement was investigated at 30 °C as no CF release occurred at this temperature under normal conditions. The effect of ethanol was investigated by stepwise addition of small quantities of ethanol (40 μL in each step) to a spectrophotometric cuvette containing a sample of liposomes containing CF. A stepwise release of CF from the liposomes was observed (Figure 3A) after the addition of each ethanol aliquot. Ethanol is known to cause change in the fluidity of the lipid membrane thanks to its incorporation into the membrane.^38^ These findings lead to the higher permeability of the membrane.^39^ The increment in CF release in each step corresponds to the liposomes whose membrane integrity was disrupted by ethanol addition. It should be noted that by adding ethanol to the sample, the local ethanol concentration at the point of addition was temporarily higher than the asymptotic average due to imperfect mixing. The effect of mixing was quantified as well, as shown in Figure S3 (Supporting Information).

The second studied permeation enhancer was oleic acid. Oleic acid incorporates itself into the membrane structure, slightly disrupts the ordered packing of the phospholipids, and makes the membrane more permeable to all molecules.^200^ Even though the measured permeation was very slow (CF release occurred over 10 h), permeability still increased from a limiting value close to zero to 6.3 × 10^–10^ cm/s (Figure 3B). The two permeation enhancement experiments demonstrate the ability of the liposomal assay to capture the effect of additional chemical species on the permeation rate of the fluorescent probe.

### Membrane Interactions Revealed by Copermeation Experiments

Having established that the liposome permeation assay makes it possible to directly observe permeation enhancement, we pose the question of whether commonly used pharmaceutical compounds might inadvertently modulate the membrane permeability or partitioning of another substance. A panel of six clinically approved drugs spanning all four biopharmaceutics classification system (BCS) classes^40^ has been chosen for copermeation experiments (Table 1). Based on their lipophilic/hydrophilic character, the drugs were incorporated into liposomes either by the aqueous route (i.e., dissolved in the hydration medium together with CF) or by the lipidic route (i.e., dissolved in chloroform and methanol together with the membrane lipids). For lipophilic compounds mildly soluble in water (HCTZ and NX), both loading methods were used (Table 1).

**Table 1:** Pharmaceutical Compounds Evaluated in Copermeation Experiments, Their Properties, and Concentrations Used^a^

**name and acronym**	**indication**	**BCS class**	**properties**	**liposome incorporation route and concentration**
ascorbic acid (ASC)	essential vitamin	class I	well soluble well permeable	aqueous (15 mg/mL)
hydrochlorothiazide (HCTZ)	hypertension	class II	mildly soluble (0.72 mg/mL^41^) well permeable	lipidic and aqueous (0.5 mg/mL)
kanamycin (KM)	antibiotic	class III	well soluble poorly permeable	aqueous (15 mg/mL)
norfloxacin (NX)	antibiotic	class IV	mildly soluble (0.28 mg/mL^42^) poorly permeable	lipidic and aqueous (0.2 mg/mL)
candesartan cilexetil (CC)	hypertension	class II	poorly soluble well permeable	lipidic (0.5 mg/mL)
apixaban (APIX)	anticoagulant	class IV	poorly soluble poorly permeable	lipidic (0.5 mg/mL)

a Note that the CF concentration was 7.5 mg/mL in all cases.

**Figure 4:**
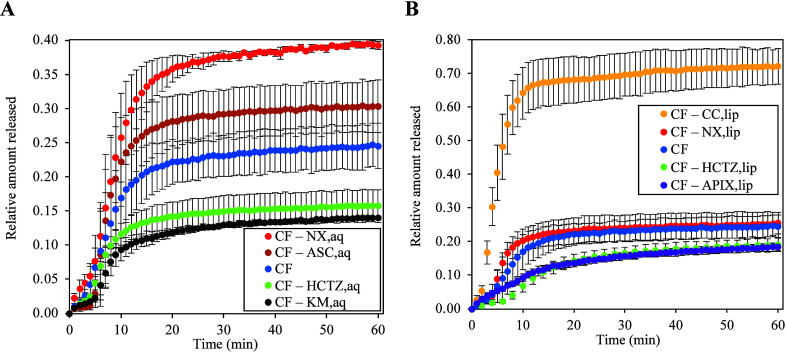
Relative amount of CF released as function time in binary copermeation experiments conducted using the liposomal assay at 40 °C. (A) Substances incorporated into liposomes by the aqueous route. (B) Substances incorporated into liposomes by the lipidic route. The permeation of CF alone is shown in both cases for reference. The acronyms of individual substances are given in Table 1. The data points are mean values; the error bars represent standard deviations (*n* = 3). Note the difference in the *y*-axis scale between cases (A) and (B).

Unexpected phenomena were observed during binary copermeation experiments (Figure 4). All investigated pharmaceutical substances (regardless of their molar weight, aqueous solubility, lipophilicity, or BCS class) had a manifestable and sometimes very strong effect on CF permeation, although these substances are not *a priori* meant to act as permeation enhancers or retardants, and no such behavior has been reported for them before. An increase in the asymptotic quantity released of CF was found for binary copermeation with ASC_aq_, NX_aq_, and CC_lip_, whereas a decrease was found for HCTZ_aq_, KM_aq_, HCTZ_lip_, and APIX_lip_ (Figure 4). Curiously, the increase in the relative amount released was caused by a pair of substances from exactly opposite BCS classes: ASC with high solubility and high permeability and NX with low solubility and low permeability. The same was true for the two substances that reduced the relative amount released: HCTZ with a low solubility and high permeability and KM with a high solubility and low permeability. These results suggest that the solubility/permeability of the copermeating substance alone is insufficient to determine its effect on the quantity released of the fluorescent probe. Clearly, both antagonistic and synergistic effects between the permeants exist, and these are sufficiently strong to change CF membrane partitioning 2–5× in both directions and permeability up to 2× upward and up to 6× downward (Table 2). From the point of view of pharmacokinetics, such changes due to drug-membrane interaction could have dramatic therapeutic implications and could potentially lead to incorrect prescription and dosing decisions, which are typically made on the assumption that each drug behaves as if it were in the patient’s body alone. As no simple rule based on the BCS class can explain the experimental data, let us briefly consider the specific features of each permeant.

**Table 2:** Experimentally Determined Values of Permeability and Partition Coefficient for CF Alone and in Copermeation in Binary Mixtures with Selected Drugs Added to the Liposomal Assay Either by the Aqueous or Lipidic Route

**sample**	**permeability(cm/s)**	**partition coefficient**
CF alone	(1.4 ± 0.4) × 10^–8^	(2.9 ± 0.2) × 10^4^
CF–ASC_aq_	(2.4 ± 0.7) × 10^–8^	(2.5 ± 0.3) × 10^4^
CF–HCTZ_aq_	(1.5 ± 0.3) × 10^–8^	(6.3 ± 0.9) × 10^4^
CF–KM_aq_	(1.2 ± 0.3) × 10^–8^	(7.0 ± 0.2) × 10^4^
CF–NX_aq_	(2.3 ± 0.5) × 10^–8^	(1.7 ± 0.1) × 10^4^
CF–CC_lip_	(2.2 ± 0.7) × 10^–8^	(1.1 ± 0.3) × 10^4^
CF–APIX_lip_	(3.1 ± 0.4) × 10^–9^	(3.1 ± 0.2) × 10^4^
CF–HCTZ_lip_	(1.1 ± 0.1) × 10^–8^	(4.9 ± 0.5) × 10^4^
CF–NX_lip_	(2.2 ± 0.2) × 10^–8^	(3.8 ± 0.5) × 10^4^

**Figure 5:**
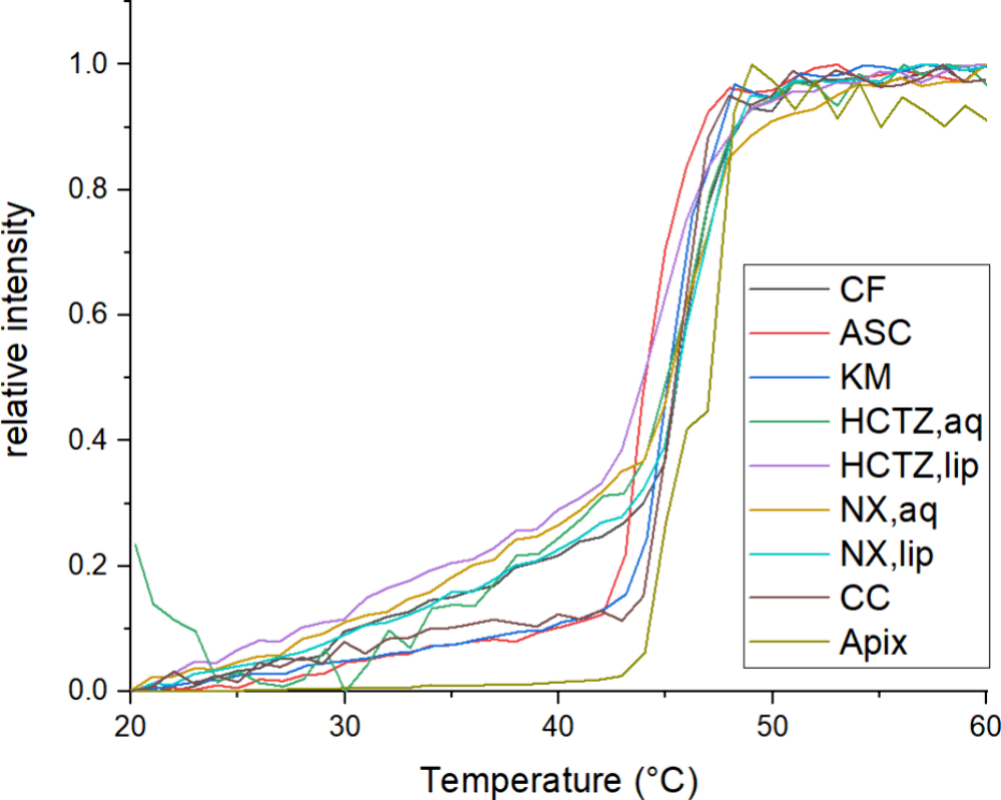
Dependency of fluorescence intensity on the temperature for pure CF samples and CF samples with individual copermeants. The relative fluorescence intensity is normalized to the maximum fluorescence intensity reached during the measurement, and the temperature scan rate was 2 °C/min.

Furthermore, to study the influence of the added copermeant on the phase behavior of the membrane, a temperature scan was performed. In this experiment, the temperature of each sample was increased by 2 °C per minute and the fluorescence intensity was measured. Figure 5 shows the results of this experiment. Temperature-dependent phase behavior of majority of the samples is close to each other. In the case of APIX, the release of CF from the liposomes is considerably lowered in temperatures before the phase change, and also, the temperature of phase change is shifted to higher values by a few degrees.

To prove that copermeants are still present in the sample after column separation and to show the concentration effects, HPLC analysis of three copermeants in final samples was performed (Table 3). CC was present in the final sample nearly in the same amount as was inserted. This shows that lipophilic drugs are bound to the membrane and stay inside. For the copermeants that were added by both ways (aqueous and lipidic), NX, HCTZ, and slight differences were observed in the final amounts. This could answer why there are different partition coefficients for HCTZ_aq_ and HCTZ_lip_; since the partition coefficient was increased in both copermeation experiments, only the value was different. In the case of NX, where there is an increase in the CF partition coefficient for NX_lip_ and decrease in NX_aq_, the answer for this behavior was not found.

**Table 3:** Analyzed Quantity of Copermeants after Liposome Separation

**sample**	**starting quantity of copermeant**(μg/mg_lipids)	**measured quantity of copermeant after column separation**(μg/mg_lipids)
CF–HCTZ_aq_	100	1.3
CF–NX_aq_	40	6
CF–CC_lip_	100	90
CF–HCTZ_lip_	100	1.0
CF–NX_lip_	40	7

**Figure 6:**
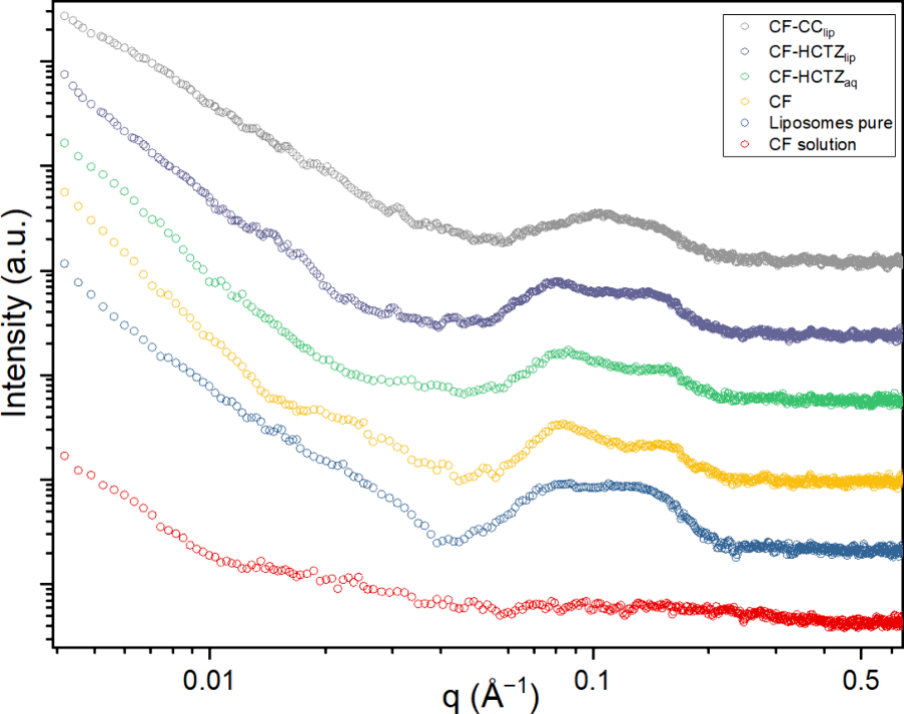
SAXS measurement of liposomes loaded by copermeants and controls. CF solution without liposomes does not exhibit any visible peaks at around 0.1 Å^–1^. Pure liposomes are liposomes hydrated by PBS only, without CF or copermeants. Other curves correspond to the respective liposome compositions, as shown in Table 2.

Small-angle X-ray scattering (SAXS) measurements were performed to study the lamellarity of prepared and purified liposomes. Varying lamellarity between prepared liposomes could affect the interpretation of the permeability and partition coefficient measurements. The SAXS curves are presented in Figure 6. A lack of a Guinier plateau in the SAXS profile and a decrease in the scattering intensity at the low *q* region following a power law of *I*(*q*) ∼ *q*^–*D*^ denotes the presence of large particles with overall sizes beyond the resolution limit (>100 nm).^43^ This perfectly corresponds to the PSD and TEM images (Figure 2) where the liposomes have an average size between 120 and 190 nm. For all samples except the negative control (CF solution), two wide peaks are observed around *q* = 0.077 and 0.143 Å^–1^. The *q* ratio of the peaks is close to 1:2, which is typical for a layered structure. Low intensity and significant broadening of the peaks suggest the unilamellar structure of liposomes^44^ since the multilamellar would have sharp peaks in this region.^45^ The lamellar spacing calculated from the position of the first-order peak according to the equation  is 8.0 nm. All of the measured SAXS curves are qualitatively comparable except for the CF-CC_lip_. This is probably caused by the incorporation of strongly lipophilic CC into the structure of the membrane, which then affects its peak positions in SAXS measurement. Nevertheless, also, this sample can be considered unilameller.

## Discussion

Ascorbic acid (ASC) was added only by the aqueous route and caused CF permeability to be approximately doubled, while the partition coefficient remained the same within the measurement error. Ascorbic acid is predominantly present in the anionic form (Table 4). Therefore, we suggest that this permeability increase can be influenced by the molecule charge. The reasoning for this can be a tilt of head groups that can occur in the presence of ions.^46^ Thus, we hypothesize that the negatively charged ASC molecules can have a similar effect: locally increasing the distances between the polar heads of the lipid molecules and therefore increasing the permeation rate of CF through the membrane without affecting its partitioning coefficient. Thus, copermeation with ASC has an enhancing effect on CF permeation.

**Table 4:**
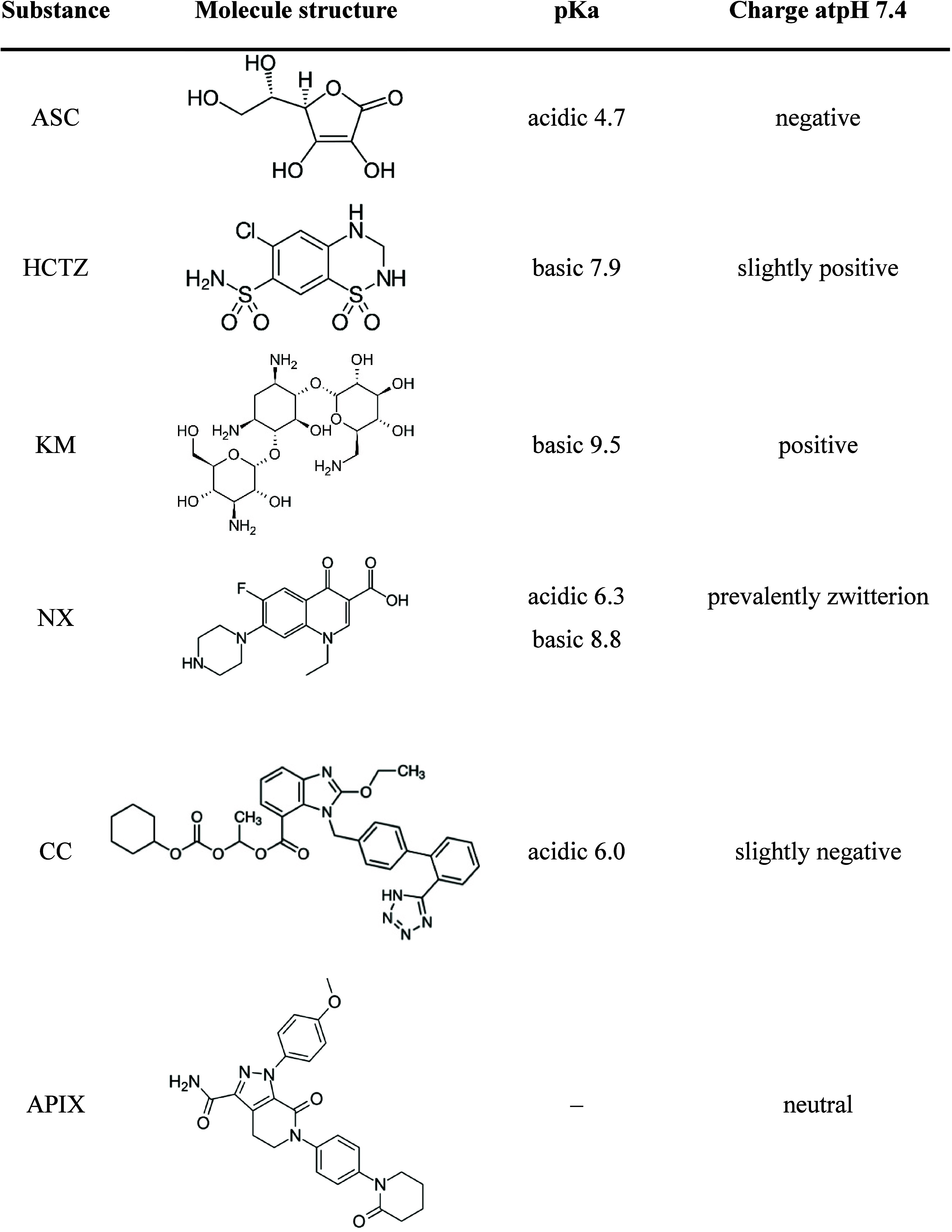
Properties of Substances Used during Copermeation Experiments with CF

Hydrochlorothiazide (HCTZ) and kanamycin (KM) had the same effect on the permeation properties of CF (permeability remained the same within the measurement error, but the partition coefficient increased). Therefore, a similarity was sought between these substances. Both KM and HCTZ have ionizable NH_2_ groups (Table 4), which allow both molecules to exist in a slightly positively charged form at the experimental pH 7.4. Either a change in the membrane packing, or a temporary association with CF, could cause an increase in membrane partitioning. It should be noted that the increase in the CF partitioning coefficient is different for HCTZ samples made by the aqueous and lipid routes (Table 2). This could be caused by the different amounts of HCTZ remaining in the sample after liposome purification, and this was shown by HPLC analysis of both samples (Table 3).

Norfloxacin (NX) again nearly doubled CF permeability, but the change of the CF partition coefficient depends on the method of addition. At pH 7.4, NX is primarily a zwitterion, but since both the basic and acidic p*K*_a_ are close to the used pH (7.4), there is a non-negligible amount of both anionic and cationic forms. An approximate ratio of the three forms is zwitterion:anion:cation = 89:7:4. The anion can play the same role in increasing CF permeability as in the case of ASC described above. The difference in the partition coefficient for both ways of addition remains unclear.

Candesartan cilexetil(CC) occurs in a slightly negatively charged form, and the trend for enhancing CF permeability was confirmed, similarly to ASC and NX. Furthermore, there was a significant decrease in the partition coefficient. This may have been because CC is a very lipophilic and large molecule, which may have displaced CF from the membrane by its presence in the membrane during copermeation. Consequently, the partition coefficient of the CF was significantly reduced.

Apixaban (APIX) caused an approximately 6-fold decrease in permeability for CF. This could be because APIX is an uncharged rigid molecule that can incorporate into the lipid membrane and change its phase behavior. Theoretically, it could incorporate into the membrane during copermeation, increasing its rigidity and decreasing its permeability for CF. Further correlative evidence for this hypothesis is the plot of the relative amount released. When CF is mixed with this substance, the curve has no inflection point, as is the case for all mixtures with other substances. Furthermore, as can be seen from Figure 5, APIX lowers the permeation rate before the phase transition temperature and changes the position of the phase transition temperature by a few degrees. At the same time, however, its incorporation does not seem to affect the partition coefficient in any way, so its presence does not displace CF from the membrane.

In conclusion, using a permeation measurement methodology based on a liposomal assay, the permeation enhancement, or suppression during copermeation of two substances has been directly investigated. As a methodology validation after the selection of an appropriate temperature, two agents with known permeation enhancement properties due to membrane disruption were studied (ethanol and oleic acid). In the case of ethanol addition, a stepwise release of the permeant (CF) was observed. This was due to the extraction of lipids from the membrane by ethanol and the loss of membrane integrity in the affected liposomes from which CF could leak out. Oleic acid worked on a different principle, which due to its incorporation into the membrane caused gradual permeation of CF even at 30 °C, i.e., well below the phase transition of the original membrane. A mathematical model of permeation enabled quantitative evaluation of permeability and the membrane partitioning coefficient of the permeant.

The liposomal permeation assay was then used for investigating the effect of six commonly prescribed pharmaceutical substances on permeability and partition coefficient during binary copermeation experiments. The chosen substances are not meant to act as permeation modifiers, and no such behavior has been measured or reported for these molecules before. Unexpectedly, all six investigated substances were found to have a significant effect on the permeability or partitioning coefficient of the permeant. Depending on the substance, either enhancement or suppression of permeation was observed (by a factor of up to 6×). The membrane partitioning coefficient was influenced by a factor of up to 5×, again both upward and downward depending on the copermeant. There was no simple correlation between the BCS class of the investigated drug and its effect on permeation. Specific molecular interactions with the permeant (CF) and membrane lipids were therefore likely the cause of permeation modification in each case.

The liposomal copermeation assay introduced in this work is fast and reproducible. The results indicate unexpected and previously unknown drug-membrane interactions that can have far-reaching consequences for the pharmacokinetics of commonly prescribed drugs in polypharmacy patients. As both permeability and the membrane partitioning coefficient can be upregulated or downregulated several times in a manner that is difficult to predict simply from the molecular properties, this work highlights the need for systematic screening of currently prescribed drugs for interactions at the permeation and biodistribution level rather than at the metabolic level. The knowledge obtained in such copermeation screening should then lead to better-informed prescription and dosage decisions by physicians who so far rely solely on single-molecule data.

## Supporting Information Available

Calibration curve for CF; TEM images of liposomes; comparison of premixed and dropwise added ethanol as a permeation enhancer; titration of CF solution with liposomes to prove reversible fluorescence quenching (PDF)
