# Supplementary material for: Liposomal Copermeation Assay Reveals Unexpected Membrane Interactions of Commonly Prescribed Drugs
Source: Mol Pharm. 2024 Apr 29;21(6):2673–83. doi: 10.1021/acs.molpharmaceut.3c00766 (PMC11151206; doi:10.1021/acs.molpharmaceut.3c00766)
Supplement: Supplementary file 1 — mp3c00766_si_001.pdf [file mp3c00766_si_001.pdf]

Supplementary material

Liposomal co-permeation assay reveals unexpected membrane interactions of commonly prescribed drugs

Klára Odehnalová[1,‡], Martin Balouch[12,‡], Kateřina Storchmannová[3], Eliška Petrová[4], Magdalena Konefał[5], Aleš Zadražil[1], Karel Berka[3], Jiří Brus[5], František Štěpánek[1,*]

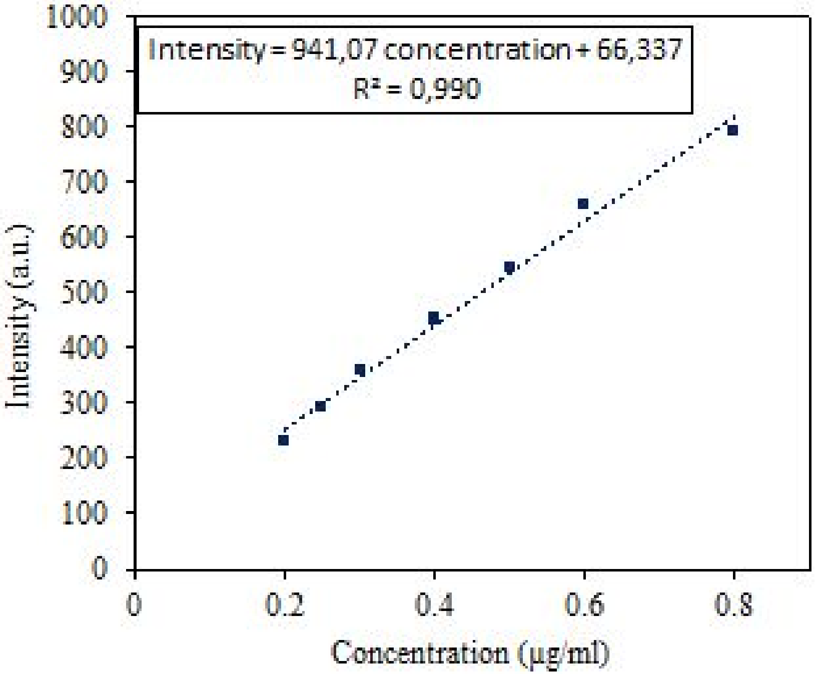

Figure S1: Calibration curve for CF. It was measured as a free CF but the possible influence of liposomes and/or Triton was also verified (always only for the lowest and the highest concentrations). Liposomes caused the fluorescence intensity in the linear region to be approximately 4 % lower. This can be caused either by the interaction of CF with the liposome membranes, or by scattering. Pure Triton added to the sample also lowered the fluorescence of CF, by approximately 7 % over the linear concentration region.

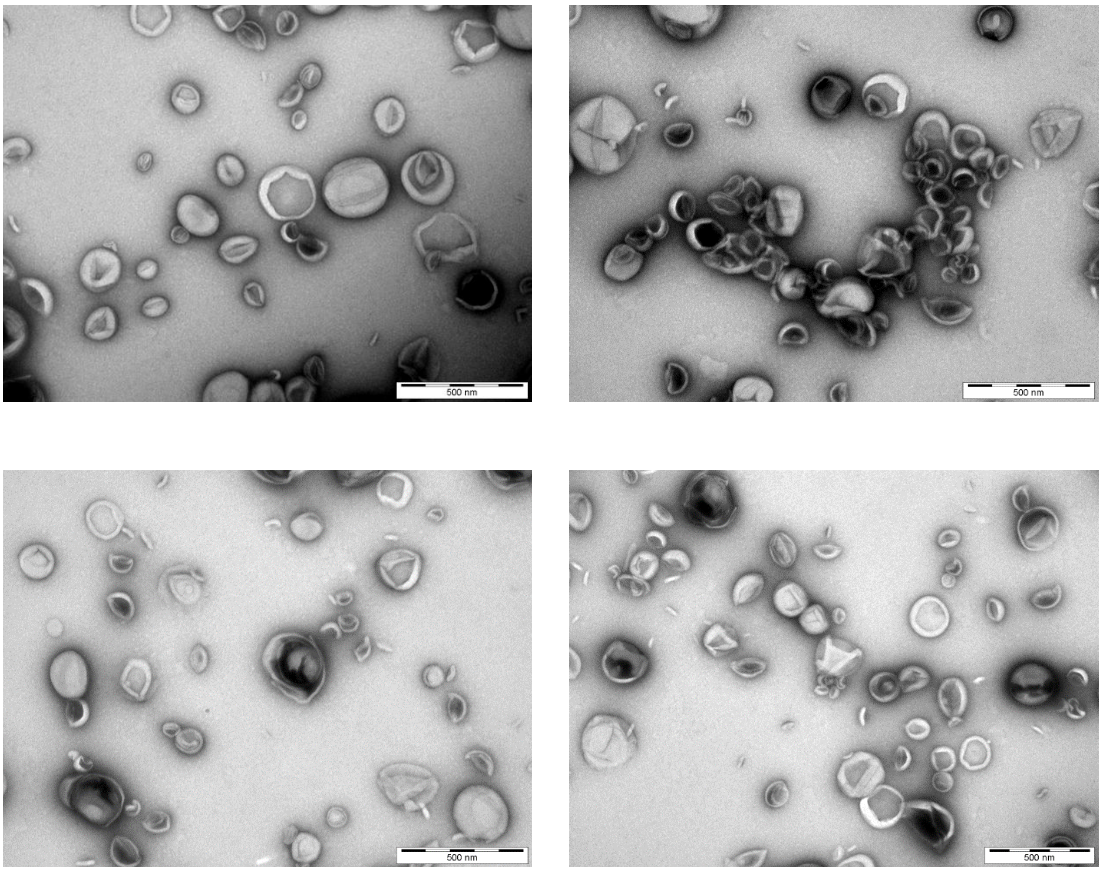

Figure S2: TEM micrographs of liposomes, showing their predominantly unilamellar structure.

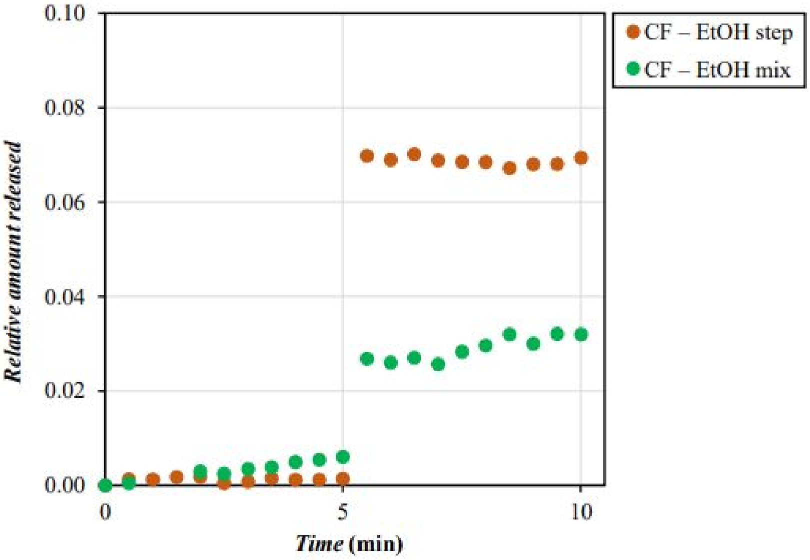

Figure S3: In experiments with ethanol and oleic acid addition, mixing was not perfect and there was a local excess of the added component at the point of addition. To quantify the effect of imperfect mixing, ethanol was premixed with PBS and only then added to the sample. A stepwise release still occurred but the jump was less steep, probably as a result of fewer liposomes being damaged.

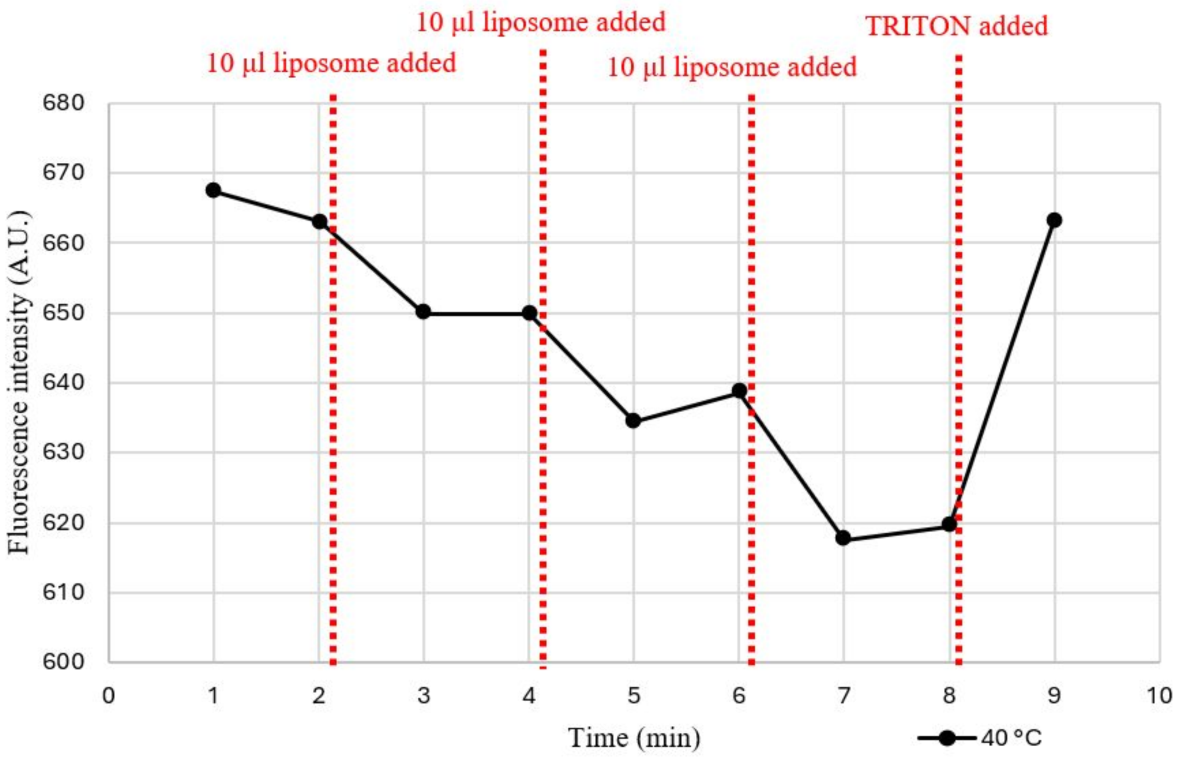

Figure S4: Titration of CF solution with liposomes at 40 ˚C to prove that the partitioning of CF into the liposome membrane results in fluorescence quenching, and that the addition of TRITON results in CF release and reversible fluorescence dequenching. A solution of pure liposomes was added to CF solution in three aliquots. This led to the lowering of fluorescence, which was proportional to the quantity of added liposomes. When TRITON was added, fluorescence intensity increased back to the original value for pure CF solution. Experimental conditions: 2000 µl of pure CF solution in PBS at pH = 7.4 (concentration 0.6 µl/mg) was used as a starting point; every 2 minutes, 10 µl of 5 mg/ml pure liposomes were added; then the sample was treated with 10 µl of 1:10 (v/v) TRITON X100.
